# Puerperal Fever—Its Pathology

**Published:** 1856-11

**Authors:** J. Boring

**Affiliations:** Professor of Obstetrics and Diseases of Women and Children, in the Atlanta Medical College


### ARTICLE V.

*Puerperal Fever—Its Pathology.* By J. Boring, M. D., Professor of Obstetrics and Diseases of Women and Children, in the Atlanta Medical College.

Few subjects have excited greater interest in the profession, and, perhaps, none have been less understood than that of Puerperal, or Child-bed Fever. And although extended thorough investigations have been had by means of hospital practice and post mortem examinations, nothing has yet been discovered by which unanimity of opinion and practice has been established. There are conflict of doctrines and disagreement in practice.

The truth of this remark is apparent, from the number and *variety* of names with which the disease has been honored, the conflicting causes assigned for its existence, and the directly opposite treatment adopted.

One calls it Puerperal Fever, another, Child-bed Fever, another, Puerperal Peritonitis, whilst others, rejecting these, divide it into Hysteritis, Ovaritis, Peritonitis, etc., as in their judgment, designating the *seat* and *nature* of the disease. Some account for its origin, by injuries sustained by the maternal organs, or placental retentions, decaying membranes, or coagulated blood, in the processes of gestation and parturition, and others hold that while these may, and often do, produce the disease, it is sometimes, especially during epidemics, caused by a *specific* virus or poison.

Such a state of the medical mind, on a subject of such moment, must be deeply deplored by all who feel the slightest interest in the science of medicine.

May we not ask, why such a diversity of opinion, why such contrariety of practice? Are these authors all right? It cannot be. Are they all wrong? We think not. There is much of truth and error intermingling in the discussion, and our manifest duty is, as far as possible, to discriminate, and avoiding the errors, avail ourselves of the light which has been shed on the subject.

That many valuable truths have been elicited, cannot be questioned, but this, like many kindred subjects, has to contend with the prejudices and prepossessions of the human mind. It is extremely difficult with men, especially those whose teachings have become *authority*, to yield an opinion, and more particularly, when to do so, implies a concession of their former errors.

Investigations, however faithfully and perseveringly conducted for the purpose of eliciting the truth, are often without the knowledge of the party, biased by opinions already formed, and as a consequence, go, in his judgement, to confirm the convictions already entertained. It is hoped, that this remark will not be construed into any, the least reflection, either upon the profession generally, or the writers referred to, particularly. We only intend, by the statement of a general truth in regard to the mind, to explain, in part, at least, how it is, that such widely different conclusions are arrived at from the very same premises.

But, notwithstanding these difficulties, much has been done in the way of discoveries, and very much of the mist and confusion by which the subject was invested, have been dissipated, and it is not unreasonable to believe that the time is not distant, when its true nature, or pathology, shall be fully understood.

Perhaps one of the greatest difficulties *yet* attending the investigation of Puerperal Fever, is that of its *name.* It will not be denied that many forms of disease, otherwise simple and easy of comprehension, have been obscured and rendered unintelligible by names, and of all these, none, we think, have suffered more than that of Child-bed Fever.

It may be seriously questioned whether it *deserves* a name. Is it a Fever ? A distinct, Idiopathic Fever ? Such as Yellow Fever, Intermittent, or is it a *Symptomatic* Fever ? We know that Fever results from inflammation of the Lung in Pneumonia, but we do not say therefore, that the patient has Pneumonic Fever. The *fever* is not idiopathic, but symptomatic, and dependent on inflammation for its existence. But, if, as we think is the fact, the Fever of child-bed arises from, and is symptomatic of, inflammation of the Uterus or its appendages, it should no more be called Fever than the same or a similar febrile action resulting from, and symptomatic of inflammation of the lung. The mere difference of location can operate no such change.

A man is thrown from his horse and breaks a leg, or suffers amputation of an arm or thigh, and fever ensues, but who would think of giving it the name of *Fever ?* A woman (an unimpregnated woman,) becomes the subject of acute Ovaritis, and takes on all the symptoms of fever, such as headache, backache, rigors, chill, greatly accelerated action of the heart and arteries, &c., but who would think of saying, she has the fever ? *Ovarian Fever ?* But why not say she has Ovarian Fever ? Because the fever is symptomatic, and is the result of local inflammation. It is not idiopathic. So we think of Puerperal Fever. It is symptomatic—it is the result of local inflammation.

Here the question naturally comes up, "is Puerperal, or Child-bed Fever, an Idiopathic Fever ?"

Those who take the affirmative, tell us, that there are two forms of the disease, one dependent on inflammation, arising from injuries done the maternal organs, in the processes of gestation and parturition, or by the retention of placental portions, or membranes, etc., and the other, upon some *virus* which, as we suppose, is in the atmosphere of the patient. But, in all candor, is not this in itself an incongruity? Is it scientific? If both these forms of Puerperal Fever exist, (which is seriously doubted,) can both be set down as Childbed Fever? Is not *one* at least, symptomatic?

If the views here controverted be correct, then, as it appears to us, at least, it would be right to say of the Pneumonic patient, "he has the '*Fever*,' 'Pneumonic Fever.'"

But it will be said, the case is not fever, but inflammation of the lung, and the fever is but a symptom. So say we of *Puerperal Fever.* The patient has inflammation of the uterus or its appendages, and hence the fever.

We are told, however, in support of the doctrine here noticed, that whilst it is true, that there is a form of the fever dependent on local inflammation, and requiring a strongly antiphlogistic treatment, there is another arising from a deadly virus, in which the vital forces are greatly depressed, and in the treatment of which, we are compelled to rely on the most powerful stimulants and tonics at our command. It is held that this form of the disease, being the very converse of the other, furnishes demonstrative proof of its dependence on a wholly different cause.

To this we reply, that the very same thing occurs in many other forms of disease, and in the case of the same disease and patient during the progress of a single spell of sickness, and yet we never for one moment think of attributing the changes to the supervention of a new and different specific cause.

We find a patient to-day, the subject of strong reaction, and put him on the antiphlogistic treatment, but tomorrow he is depressed, prostrate, and requires a different treatment altogether, and we pass through the whole, nothing surprised, nor one time supposing that we had mistaken the form of disease, or that a new form had supervened. We *know* that on yesterday the disease was at a given *stage*, and to-day it has reached another, in which the symptoms are modified accordingly. Take, for illustration, a case of Pneumonia.

We are called to the patient in the first *stage* of the disease, and find head ache, back ache, thirst, a bounding pulse, pain in the chest, laborious, hurried breathing, and, in a word, a high degree of excitement, constituting fever. Here we have Pneumonia, and the indication is, to reduce the excitement, which we attempt by the lancet, antimonials, etc. A day or two hence, on visiting our patient again, we find him depressed, prostrate, the vital forces stunned, and the indication inferred is, to sustain, by wine and other stimulents, and by powerful tonics. *But we still have Pneumonia.* It never enters the mind that this *must* be a different disease, because we have a new class of symptoms. It is Pneumonia in the second *stage*, and not a new disease. Suppose, however, we should not be called in until the supervention of the second or third stage, and find on our first visit, the patient perfectly prostrate, and upon an examination of the case, detect an abscess in the lung, and the tendencies to prostration to resist all our remedies. Shall we conclude that there are two forms of Pneumonia, dependent each on its own cause? That the one is Inflammatory, and the other Typhoid? We think not. Such are the facts in Puerperal Fever. To us, at least, the two cases seem, in this respect, exactly analagous.

Puerperal Fever has its inflammatory, its suppurative and gangrenous stages, and remembering, as we should, the nature of the tissues and organs involved, it should not seem strange that the transitions should sometimes be so rapid as to elude our most scrutinizing investigations. Indeed, we should remember that an inflammation, involving, as this sometimes does, the uterine system, together with most or all the Peritoneum, may often utterly overwhelm and stun the nervous system, in the course of a few hours, so that on our first visit to the case, we should find *extreme* nervous symptoms, &c., demanding stimulants and tonics.

With these obvious facts before us, we can see no necessity for resorting to the idea of a concealed virus, to account for the results. The nature and extent of the inflammation are quite sufficient for the effects. That numerous circumstances may exert a modifying influence on disease, and this, as others, none will doubt. Constitution, age, habit, climate, cold, attendant circumstances, &c., may, each and all, modify, but not change the character of the disease.

With the knowledge of this law of disease, should it seem strange that parturient patients, crowded together, as they often are, in the lying-in hospitals of Europe, breathing, as they must, the air already impoverished by having been so often appropriated by others, and impregnated by their impurities, should suddenly collapse, and seem as if some fatal blow from an unseen arm had stricken them down? Is it not rather just what should be expected?

It will have been seen from the foregoing, that we do not believe the disease under investigation entitled to the name of *fever*, and consequently hold that the fact that authors have classed it as a distinct form of idiopathic fever has served to obscure it, and lead the mind astray on the subject of its true pathology.

Holding, as we do, that Puerperal Fever is nothing more nor less than inflammation of the uterine system, or some portion of it, or of the Peritoneum, or of the whole, as may happen in any given case, we cannot see why it should receive the name of fever any more than any and all other inflammations *producing* fever as a symptom.

That the pathological character of Child-bed fever is that of inflammation, seems to us indisputable, yet as there are those, and indeed many who differ with us on the subject, it will not be irrelevant to adduce *some* at least of the reasons by which our conclusion is reached.

We cannot claim, as some, to have enjoyed frequent opportunities of post mortem examinations on this subject, but we have the reports of those who *have*, and rely as fully on them for accuracy and truth, as though we had seen with our own eyes, and handled with our own hands, and this we do the more freely, since *all agree* that there are unmistakable signs of inflammation. We say, all agree touching this fact, the only difference worthy of remark being found in the fact, that the advocates of the doctrine of two forms of the disease, claim that there is a form in which the traces of inflammation though manifest, are not in proportion to the malignity of the attack, and that there *must* have been in such cases, a more virulent *cause*.

Although we think the differences of the disease under different circumstances, and at different *stages* have been sufficiently accounted for, we hope it may not be uninteresting to make a further remark at this point in the discussion.

Keeping before us the fact, that the disease is often greatly, and indeed *strangely* modified by circumstances, we ask indulgence while we state that it is by no means clear to us that Puerperal Fever, or the pathological condition *called* Puerperal Fever, does not sometimes assume other aspects, and present other developments, to which, hitherto, the attention of the profession has not been called.

We are not prepared to assert, but *suggest*, that it may not be impossible, that phlegmasia dolens and mammary abscess are but other developments of this pathological condition, and in common with what is called Puerperal Fever, dependent on uterine derangement for their existence.

Strange and unorthodox as this suggestion may seem at the first presentation, it is hoped the author may not be condemned until heard in his defence. The first thought of any possible connexion between Child-bed Fever and Milk-leg, was suggested to us, by an observance of the fact, that in the incipiency of these diseases—the pathological condition of the patient is in every respect precisely the same. So true is this remark that when in charge, or called to a case in the early stage, (say the formative stage,) we have been compelled to wait further developments, to determine whether we were to have a case of Milk-leg or fever. In fact, it should be further stated, that in this stage of the disease, it is impossible to say whether we are to have a normal secretion of milk, mammary inflammation, Milk-leg *or* Puerperal Fever. We have headache, shivering, rigors, perhaps an ague, succeeded by fever, while there is tenderness in the pelvic or abdominal region, which never fails to excite the interest of the observing physician, and which state of things every physician knows, may result in the secretion of milk or mammary inflammation, in milk-leg or puerperal fever. We have noticed, too, that in fever and milk-leg, there is generally about the same interruption of the lochial discharge, derangement manifest of the womb, and tenderness both in the uterine and ovarian regions, particularly the ovarian.

Further, we have remarked that any one of these developments seems to substitute the others. When we have milk-leg, puerperal fever and mammary abscess are absent, and when we have abscess or fever, milk-leg is absent. We do not assert that this is always the case, but cannot now call to mind an exception to the rule. It is also well known, that milk-leg and puerperal fever greatly modify or arrest the secretion of milk by the mammary glands, thus demonstrating the intimacy and vitality of the relations of these organs. In harmony with the foregoing, it is in place to state that during the year 1855, we observed in this city a strong tendency in parturient patients, to develop child-bed fever, and in some few cases, milk-leg, but no mammary abscess. In our practice the present year (1856), there has not occurred a case of puerperal fever, whilst mammary abscess has been frequent and troublesome. This may all have happened to us, without any such influence as that to which we refer it; but the conviction is strong that there is more of truth than speculation involved.

That metastasés more remarkable do occur in the practice of every physician, all admit; we know, too, that so remarkable are the connexions between the uterus and mammary glands, that the one is constantly affected by the other.

May we not also appeal to the well known fact of vicarious menstruation in support of these views. In this case, by some mysterious law, when the uterus is disabled by disease, so as not to perform its singular function, menstruation, the eye, the nose, the breasts, the stomach, bowels, lungs, and even an ulcer, will take on the uterine office. Is it less rational to suppose, that in harmony with the law of metastasés, the transfer of disease from one to another organ may take place, and that its existence in one may protect another from its invasion?

It is a fact long since established, that protracted derangement of menstruation will superinduce pulmonary disease, perhaps, tubercular deposits and Phthisis Pulminalis itself, by which is demonstrated the certain and powerful influence of uterine disease upon other and distant organs, a fact which can never be too well understood and appreciated. But we may not pursue this subject further at present.

For the correctness of the remark, that post mortem examinations have demonstrated the existence of inflammation in the uterine system, or peritoneum, almost or quite uniformly, we must refer to the works of those who have written on the subject. Having already swelled this discussion far beyond our first intention, we fear to intrude further, on the patience of the reader. It is especially requested that the statements and remarks of that deservedly distinguished American author, Professor Meigs, be carefully read and weighed. They claim the highest consideration at the hands of American physicians. See the chapter on "Child-bed Fever," in his work on Obstetrics. In further confirmation of these views, of the pathology of this disease, it may, with great propriety, be asserted that all the symptoms attending it, are just such as would be looked for in such an inflammation as is contended for. Let any man look at the nature, extent and probable effects of the inflammatory action set up, and he will expect the whole catalogue of symptoms so graphically described by our authors.

The causes, too, to which we assign the origin of this pathological condition, seem to us quite sufficient for its production, and in so far as they correspond, to establish the doctrine. We say it is inflammation of the uterus, its appendages, or both, or of the peritoneum, or sometimes of the whole—we say in confirmation, that post mortem examinations have detected unmistakable traces of inflammation—that all agree to this statement—that the symptoms are just such as should be expected in such an inflammation—that the causes to which this pathological condition is assigned, are adequate to the production of such an effect—that the uterus, from its normal state to the end of gestation, and from the end of gestation, through parturition, and its sequences, to the *resumption* of its normal condition, is impelled through changes, vast and rapid, by which, from the nature of things, it is, and *must be exposed to inflammatory action*, to say nothing of the numerous incidental circumstances, as mal-practice in Obstetrics, cold, vitiated air, &c., which may conspire to assail the already enfeebled organ, it seems to us undeniable. And let it not be said, that this argues defect in the works of nature, since gestation and parturition are normal operations. It is true, they are normal, *but it is likewise true, that every operation of nature is liable to incidental evils, as results from normal action.*

Mastication of the food is a normal action, but the very process wears away the teeth, and he who eats must expect the consequences.

The secretion of milk, and suckling a child, by a recent mother, are normal operations, but she who secretes and suckles, may have excoriated nipples and mammary abscess as the results.

